# Effect of Trehalose/OEO/Tween 80/Tween 20 Addition on Physical Stability of Edible Packaging during Storage in Different Humidity Conditions

**DOI:** 10.3390/foods12152903

**Published:** 2023-07-30

**Authors:** Simona Dordevic, Dani Dordevic, Karolina Tesikova, Bohuslava Tremlova

**Affiliations:** Department of Plant Origin Food Sciences, Faculty of Veterinary Hygiene and Ecology, University of Veterinary Sciences Brno, Palackeho tr. 1946/1, 612 42 Brno, Czech Republic; dordevicd@vfu.cz (D.D.); tesikovak@vfu.cz (K.T.); tremlovab@vfu.cz (B.T.)

**Keywords:** edible/biodegradable packaging, environment humidity, textural properties, water solubility

## Abstract

Edible packaging has been a topic of much discussion during recent years, mainly due to its lower environmental impact. This study aimed to investigate the properties of an edible packaging made from a combination of carrageenan, orange essential oil (OEO), and trehalose (Tre) under different humidity conditions. The films were analyzed based on their water content, solubility, and textural properties, such as strength and breaking strain. The results of the study showed that the addition of trehalose reduced the water content and increased the strength of the packaging, regardless of the humidity conditions. The inclusion of orange essential oil also contributed to lower water content, which led to more water-resistant packaging (during standard humidity conditions (45%)—c: 15.31%; Tre3OT80: 4.04%, Tre1OT80: 4.48%). The findings of this study have important implications for the production of stable and environmentally friendly edible packaging. The results demonstrate the potential of trehalose and orange essential oil as additives to enhance the properties of edible packaging, particularly in terms of its resistance to moisture. The best results were found in Tre1OT80 and Tre3OT80 samples. Moreover, the study emphasizes the importance of considering storage conditions in the development of edible packaging, as different humidity levels can significantly affect the packaging’s properties and shelf life. The findings have practical applications for the food industry, particularly in the development of sustainable packaging solutions and for further studies where the application of this packaging can be analyzed for different foodstuffs.

## 1. Introduction

Edible/biodegradable packaging is important in lowering environmental pollution caused by synthetic packaging materials. Edible packaging consists of polysaccharides, proteins or lipids [1]. It is a well-known fact that edible packaging is typically composed of natural polymers such as polysaccharides, proteins or lipids, which serve as the basic matrix of the material. However, this basic composition can be further enriched and enhanced by the addition of various natural extracts, essential oils, and other complementary combinations, providing an even broader range of properties and potential applications for these innovative packaging solutions [2].

Carrageenan is very commonly used as a base matrix for the production of edible/biodegradable packaging. It is a linear sulfated polysaccharide derived from red algae. Carrageenan is also very widely used in various branches of the food and pharmaceutical industries. Carrageenan is described in three different forms, κ, ι and λ, which are distinguished by differences in structure: different content of 3,6-anhydrogalactose and the position and number of ester sulfate groups [3]. The essential oils are incorporated into edible packaging because they have antimicrobial, antioxidant and bio preservative effects [4].

Trehalose is a non-reducing disaccharide. It can be found in many natural sources such as mushrooms, honey or bakery yeasts [5]. The advantage of trehalose is to protect proteins from chemical reactions: denaturation and inactivation. These reactions can be caused by dehydration, cold, heat, desiccation or oxidation. Due to this property, Tre can protect foods during frozen storage [6,7].

It is important to analyze these types of packaging before they are applied to food, especially their properties under different conditions, such as different humidity conditions, which can affect their structural properties. Undoubtedly, the strength of the packaging is a vital and highly significant parameter that plays a pivotal role in ensuring the protection and preservation of the packaged food against any potential external influences. This essential characteristic is essential in ensuring that the food products remain fresh, intact and safe for consumption during storage, transportation and distribution. It is, therefore, a key consideration in the development and evaluation of any packaging material or system [8,9].

Usually, the most studied parameter of edible packaging in different humidity conditions is oxygen barrier properties [10], but other properties are important too, for example, textural properties and other physical properties. While there are some studies addressing the effects of moisture on molded fiber and paper packaging [11,12], remarkably, to date, there has been an absence of any published articles or research studies specifically addressing the issue of the potential effects of varying moisture conditions on carrageenan-based packaging, despite the growing interest and demand for edible packaging solutions. This highlights the critical need for further research in this area, which could provide valuable insights into the potential limitations and optimization opportunities for carrageenan-based packaging under different humidity conditions, and ultimately contribute to the development of more effective and sustainable packaging solutions.

This research presents an innovative approach to edible packaging by investigating and emphasizing the following issues: (a) the properties of a novel combination of carrageenan, orange essential oil (OEO) and trehalose (Tre) under varying humidity conditions; (b) this edible packaging offers a low environmental impact, making it an attractive option for sustainable packaging solutions; (c) the study also emphasizes the importance of considering storage conditions, as different humidity levels significantly influence the packaging’s properties and shelf life; (d) the research has significant implications for the food industry, offering stable and environmentally friendly packaging alternatives; and (e) the study paves the way for future studies focusing on the application of these innovative packaging materials to various foodstuffs, broadening their potential use and impact in the industry.

This study follows up on the previous study by Jancikova et al. [13]. The edible packages composed of κ-carrageenan, OEO, and with different concentrations of Tre were prepared and stored under different humidity conditions. Subsequently, water content, solubility and textural property analyses were performed to improve the knowledge of how these properties change under different humidity conditions.

## 2. Materials and Methods

### 2.1. Materials

The κ-carrageenan, Tween 20 and Tween 80 were obtained from Sigma Aldrich (St. Louis, MO, USA). Trehalose was purchased from Toppotraviny.cz s.r.o. (Prague, Czech Republic). The orange essential oil was obtained from the local market (DM-drogerie markt GmbH&Co KG, Brno, Czech Republic).

### 2.2. Preparation of Different Humidity Conditions

The humidity conditions were prepared by the saturated solutions of the following salts: MgCl_2_, NaCl, NaBr and KNO_3_, which were put in the desiccator for 5 days for humidity stabilization. The salts were chosen based on the reported humidity—MgCl_2_ × 6H_2_O—32.8%; NaBr—57.6%; NaCl—75.3%; KNO_3_—93.6% [14]. Silica gel was used for the 0% humidity conditions.

### 2.3. Preparation of Edible Packaging

The composition of edible packaging is described in Table 1, according to Jancikova et al. [13]. The edible films consisting of carrageenan and trehalose were prepared as follows: The κ-carrageenan and trehalose (0.5; 1 and 3%) were weighted in a beaker and 45 mL of distilled water was added. The carrageenan and trehalose were dissolved and then heated. The samples were then put on a magnetic stirrer and stirred for 10 min. Then, 0.25 mL of glycerol was added, and after 5 min the film-forming solution was cast in a Petri dish (diameter 9 cm).

The edible films consist of carrageenan, trehalose and orange essential oil. The carrageenan and trehalose were added into a beaker. The distilled water was added. The carrageenan and trehalose were dissolved. The beaker with the solution was heated and then put on a magnetic stirrer for 10 min (350 rpm). Afterwards, 0.45 mL of orange essential oil was added, followed by 5 min of stirring. Then, 0.25 mL of glycerol was added along with 1.3 mL of Tween 20 or Tween 80. The film forming solution was cast on Petri dish (diameter 9 cm) and dried.

### 2.4. Storage of Packaging

The prepared edible films were stored in desiccators for 4 days. The analyses were performed directly after removing the films from the different humidity conditions. Except for the humidity conditions described in Section 2.2 (Preparation of different humidity conditions), the edible films were also stored at laboratory humidity conditions (45%). All films were stored at the laboratory temperature.

### 2.5. Water Content and Solubility

Water content was analyzed by the method by Kavoosi et al. [15] and Souza et al. [16], with slight modifications. The films were cut to 2 × 2 cm diameter and weighted (W1). Then, the films were put in the laboratory oven (Ecocell 55, BMT Medical Technology s.r.o., Brno, Czech Republic) for 2 h at 105 °C. Afterwards, the films were weighted again (W2). Subsequently, the films were put in 25 mL of distilled water and after 24 h, the films were removed and weighted (W3). The water content and solubility were calculated according to the following formulas:

Water content (%) = (W1 − W2/W1) × 100(1)

Solubility (%) = (W2 − W3/W2) × 100 (2)

### 2.6. Textural Properties

The strength (MPa) and elongation at break (%) were measured by texturometer TA.XT plus (Goldaming, UK) by ASTM method number D882-02. The edible films were cut to 1 × 5 cm and analyzed five times.

### 2.7. Statistical Analysis

The results were statistically analyzed using IBM SPSS 20 software. One-way analysis of variance was used. The statistical significance at *p* < 0.05 was evaluated. A nonparametric Games–Howel post hoc test and parametric Tukey post-hoc test were used to analyze the difference within groups. PCA (principal component analysis) was used for the determination of difference among all analyzes and samples.

## 3. Results and Discussion

### 3.1. Water Content and Solubility

The results of water content are summarized in Table 2. When comparing the results, it was found that regardless of the humidity conditions, samples c, cTre0.5, cTre1, and cTre3 had the highest water content. These film types did not contain essential oil or Tween 20 or 80. However, with the addition of trehalose, the water content decreased, which can be attributed to the low hygroscopicity (in addition to mild sweetness, lower glycemic index, low carcinogenicity, and protein protection properties) of the dehydrate crystal of trehalose. It is also known that low hygroscopicity of trehalose results in stronger protein–sugar bonds [7,17].

These findings may help in the production of edible packaging that is more stable under higher humidity conditions [18,19] because when the trehalose concentration was higher, the water content was lower under all humidity conditions tested. It has been demonstrated that an elevated concentration of trehalose, a naturally occurring disaccharide, yields outcomes in terms of enhancing the stability and robustness of packaging materials, particularly when subjected to challenging high humidity conditions. One of the most widely recognized and extensively studied aspects that exemplifies the multifaceted nature of trehalose lies in its remarkable capacity to serve as a protective shield, preserving not only the structural integrity but also the functionality of highly vulnerable constituents present in various food matrices, which are inherently susceptible to the adverse effects induced by dehydration [20,21]. The foremost advantages of trehalose lie in its extraordinary capacity to bind water molecules, thereby impeding the formation of ice crystals both within cellular structures and in the external environment. This unique property assumes paramount importance in the realm of freezing processes, as it serves as a safeguard, ensuring the preservation of cellular integrity and safeguarding the overall quality of frozen products. This invaluable attribute has found applications not only in the food industry but also in the pharmaceutical sector, where trehalose plays a crucial role in stabilizing delicate proteins and enzymes during intricate freeze-drying procedures [22].

It has been revealed that the incorporation of orange essential oil leads to a noticeable reduction in water content within the packaging materials. This can be attributed to the intricate interaction between the essential oil and the hydroxyl groups present in the carrageenan matrix. It is postulated that this interaction triggers a chemical reaction, resulting in a decrease in the availability of hydroxyl groups that would otherwise participate in interactions with water molecules. Consequently, the resultant films exhibit a heightened resistance to water, manifesting as improved water resistance and enhanced durability in the face of moisture-laden environments [23,24]. When orange essential oil was mixed with trehalose, the films with the lowest water content were observed. This was seen in samples labeled with the following marks: Tre0.5OT80, Tre0.5OT20, Tre1OT80, Tre1OT20, Tre3OT80, and Tre3OT20. The combination of orange essential oil and trehalose enhanced the water-binding capacity of the films, making them suitable for applications such as food packaging and controlled-release drug delivery systems.

Their solubility is not listed in Table 2, because for each sample it was found that they are fully soluble (100%) in distilled water. These findings align with previously published research [25].

### 3.2. Textural Properties

The results of the strength of stored edible packages are summarized in Table 3 for packages without added essential oils. Strength, in the context of materials science and engineering, refers to the maximum tensile stress that a material can endure before undergoing permanent deformation. It is a fundamental property that characterizes the mechanical integrity and durability of a substance. When a material is subjected to external forces or loads, such as pulling or stretching, its strength determines the point at which it will yield or permanently deform. This critical threshold is typically defined by the maximum stress that the material can sustain without experiencing significant or irreversible structural changes. Understanding the strength of a material is crucial for designing structures, selecting appropriate materials for specific applications, and ensuring their ability to withstand various mechanical forces or stresses [26]. The increasing addition of trehalose increased the strength of packaging in 0%, 45%, 57% and 74% humidity conditions.

Storage of the packages at 0% humidity showed an increase in strength with the addition of trehalose. At different humidity conditions, strength increased for samples c, cTre0.5, and cTre1; however, strength decreased for sample cTre3.

Previous research has indicated that the strength of food packaging tends to diminish as humidity levels rise. This can be attributed to the increased absorption of water by the matrices of food packaging materials, leading to a reduction in the overall strength of the stored samples. The higher moisture content in the packaging matrices weakens their structural integrity, thereby compromising the strength and durability of the packaging material, as demonstrated in previous studies [18,27,28].

The results of samples with addition of OEO are summarized in Table 4. When comparing the addition of essential oil to matrices with other samples, it was found that the essential oil reduced the strength of the packaging, probably due to the oil’s plasticizing ability EO [29]. Moreover, when the addition of different emulsifiers is compared, in almost all samples the packages with addition of Tween 20 had lower strength than the packages with addition of Tween 80, regardless of the environment humidity values.

The results of breaking strain are summarized in Table 5 for samples without the addition of OEO. There should be found, according to the previous section, that with the loss of strength the result of breaking strain should increase. However, when the packages were stored at 45% humidity and 92% humidity, elongation at breaks decreased, but only for samples cTre0.5 and cTre1 were the decreases statistically significantly different (*p* > 0.05). These findings may be caused by excessive humidity, which limits the plasticizing effect of food packaging, and this limitation may lead to the reduction in breaking strength [30].

The results of the samples with the addition of OEO are shown in Table 6. In line with the findings on fracture strength, the incorporation of orange essential oil into the experimental samples was observed to have a positive effect on the fracture strength. The addition of orange essential oil resulted in an enhancement of the fracture strength parameter, indicating improved structural integrity and resistance to breakage. This outcome suggests that the presence of orange essential oil in the samples contributes to reinforcing the overall strength of the material, potentially attributed to its unique chemical composition and interaction with the matrix components. Similar findings were found by Ghasemlou et al. [31] and Suput et al. [32]. When the addition of Tween 20 or Tween 80 was compared, there was no trend. Only when the samples were stored in standard humidity conditions (45%) was it found that the samples with the addition of Tween 20 had higher breaking strain than the samples with the addition of Tween 80. Nevertheless, establishing a definitive trend regarding the effect of increasing humidity on elongation at break proved inconclusive. While there is no clear consensus, it is generally observed that higher humidity levels tend to decrease the elongation at break of materials. The presence of increased moisture content in the surrounding environment can potentially affect the material’s flexibility and elongation properties, leading to reduced stretchability and resistance to breakage. More comprehensive understanding of the relationship between humidity and elongation at break, as other factors such as material composition and structure may also play a role in determination of the outcome.

### 3.3. Principal Component Analysis

**Figure 1:**
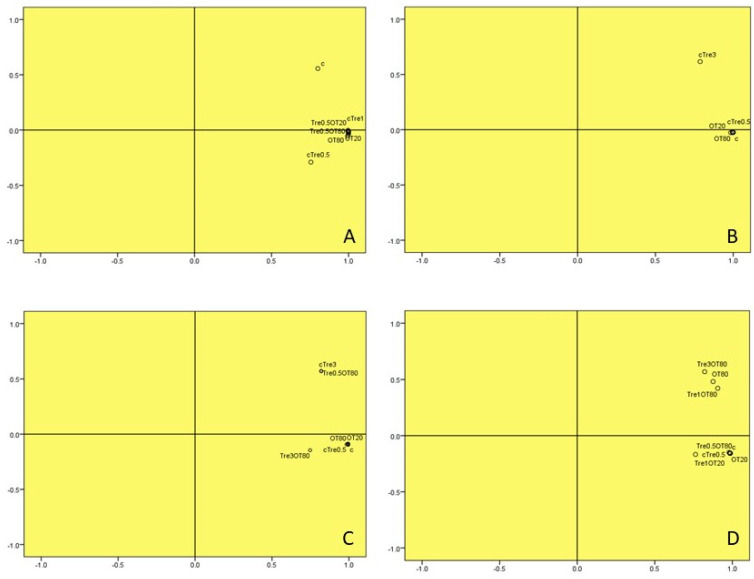

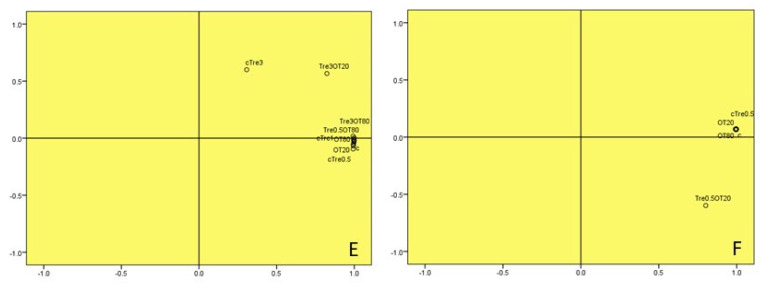
Principal component analyses for samples stored at: (**A**) KNO3 (92%); (**B**) MgCl2 (32%); (**C**) silica gel (0%); (**D**) NaCl (74%); (**E**) laboratory humidity (45%); (**F**) NaBr (57%).

The analysis of experimentally produced edible/biodegradable packaging under different humidity conditions was conducted using principal component analysis (PCA), as shown in Figure 1. The results obtained from the PCA indicated significant differences (*p* < 0.05) between the control samples and the other experimentally produced packaging samples when exposed to 92% humidity (Figure 1A). Furthermore, when comparing different humidity conditions, it was observed that CTRE3 exhibited significant differences (*p* < 0.05) compared to the other samples under 32% and 45% humidity conditions (Figure 1B,E). Similarly, the sample Tre0.5OT20 displayed significant differences (*p* < 0.05) compared to the samples under 57% humidity conditions. These findings highlight the impact of humidity on the properties and characteristics of the experimentally produced packaging, demonstrating the sensitivity of the materials to varying humidity conditions. The observed differences, as identified through the PCA analysis, provide overall insights into the influence of humidity on the performance and behavior of the edible/biodegradable packaging materials.

## 4. Conclusions

In conclusion, the addition of trehalose to edible packaging films decreased water content and increased strength, especially when stored under higher humidity conditions. The addition of orange essential oil decreased water content and increased fracture strength but reduced the strength of the packaging. The elongation at break decreased with increasing humidity, which may limit the plasticizing effect of food packaging and lead to a reduction in breaking strength. Overall, these findings may help in the production of more stable and effective edible packaging for food products. The suggestions for future studies would be the following: (a) investigation of the potential synergistic effects of combining trehalose with other natural additives; (b) long-term stability assessment; (c) impact on food quality; and (d) environmental impact analysis that would include biodegradability and waste management.

## Figures and Tables

**Table 1:** Composition of prepared edible packaging.

Sample	Composition
c	0.3 g κ-carrageenan + glycerol
OT80	0.3 g κ-carrageenan + tween 80 + orange essential oil + glycerol
OT20	0.3 g κ-carrageenan + tween 20 + orange essential oil + glycerol
cTre0.5	0.3 g κ-carrageenan + 0.5% trehalose + glycerol
Tre0.5OT80	0.3 g κ-carrageenan + 0.5% trehalose + tween 80 + orange essential oil + glycerol
Tre0.5OT20	0.3 g κ-carrageenan + 0.5% trehalose + tween 20 + orange essential oil + glycerol
cTre1	0.3 g κ-carrageenan + 1% trehalose + glycerol
Tre1OT80	0.3 g κ-carrageenan + 1% trehalose + tween 80 + orange essential oil + glycerol
Tre1OT20	0.3 g κ-carrageenan + 1% trehalose + tween 20 + orange essential oil + glycerol
cTre3	0.3 g κ-carrageenan + 3% trehalose + glycerol
Tre3OT80	0.3 g κ-carrageenan + 3% trehalose + tween 80 + orange essential oil + glycerol
Tre3OT20	0.3 g κ-carrageenan + 3% trehalose + tween 20 + orange essential oil + glycerol

**Table 2:** Water content in produced edible packages stored under different humidity conditions.

Samples	Silica gel (0%)	MgCl_2_ (32%)	Standard (45%)	NaBr (57%)	NaCl (74%)	KNO_3_ (92%)
c	6.68 ± 1.84 ^D^	18.94 ± 0.13 ^A^	15.31 ± 1.46 ^A^	17.93 ± 1.07 ^A^	30.24 ± 0.65 ^B^	55.86 ± 3.00 ^C^
OT80	4.08 ± 0.51 ^A^	7.19 ± 1.01 ^EF^	5.17 ± 0.60 ^AF^	8.61 ± 1.46 ^DE^	24.48 ± 1.02 ^B^	32.78 ± 1.02 ^C^
OT20	4.99 ± 0.39 ^A^	8.24 ± 0.44 ^D^	4.40 ± 0.10 ^A^	9.11 ± 1.58 ^D^	22.72 ± 0.42 ^B^	36.32 ± 0.69 ^C^
cTre0.5	7.76 ± 2.07 ^C^	15.26 ± 1.77 ^AC^	14.79 ± 2.17 ^AC^	17.38 ± 0.72 ^A^	17.52 ± 0.56 ^A^	48.23 ± 1.75 ^B^
Tre0.5OT80	4.58 ± 0.99 ^A^	7.73 ± 0.75 ^A^	5.02 ± 0.72 ^A^	12.85 ± 3.04 ^AD^	22.81 ± 0.93 ^BD^	32.78 ± 1.02 ^C^
Tre0.5OT20	3.90 ± 0.70 ^D^	8.43 ± 0.20 ^A^	6.35 ± 0.82 ^AD^	8.34 ± 0.13 ^A^	21.86 ± 0.29 ^B^	39.66 ± 0.61 ^C^
cTre1	6.22 ± 0.40 ^D^	13.79 ± 1.03 ^B^	10.45 ± 0.89 ^A^	13.85 ± 1.23 ^B^	16.09 ± 0.71 ^B^	45.91 ± 0.60 ^C^
Tre1OT80	4.17 ± 0.11 ^A^	6.92 ± 0.42 ^E^	4.48 ± 0.32 ^A^	10.07 ± 0.61 ^D^	19.36 ± 0.87 ^B^	29.07 ± 0.55 ^C^
Tre1OT20	4.66 ± 0.62 ^A^	6.83 ± 0.68 ^E^	5.52 ± 0.21 ^AE^	8.97 ± 0.33 ^D^	20.69 ± 0.97 ^B^	38.63 ± 0.76 ^C^
cTre3	5.18 ± 0.08 ^A^	9.29 ± 0.21 ^E^	4.93 ± 0.21 ^A^	11.56 ± 0.22 ^D^	20.62 ± 0.88 ^B^	34.08 ± 0.56 ^C^
Tre3OT80	3.43 ± 0.45 ^A^	7.62 ± 0.12 ^E^	4.04 ± 0.21 ^A^	13.62 ± 2.96 ^ADE^	17.03 ± 0.15 ^BD^	30.31 ± 0.35 ^C^
Tre3OT20	3.52 ± 0.25 ^D^	6.97 ± 0.44 ^A^	6.96 ± 0.43 ^A^	9.26 ± 0.35 ^C^	15.39 ± 0.24 ^B^	37.91 ± 8.07

Different letters indicate statistically significant differences (*p* < 0.05) between columns.

**Table 3:** Strength (MPa) of edible packaging without addition of orange essential oil.

Sample	Silica gel (0%)	MgCl_2_ (32%)	Standard (45%)	NaBr (57%)	NaCl (74%)	KNO_3_ (92%)
c	0.10 ± 0.01 ^AB^	0.11 ± 0.01 ^AB^	0.14 ± 0.03 ^A^	0.10 ± 0.01 ^AB^	0.13 ± 0.04 ^AB^	0.06 ± 0.03 ^B^
cTre0.5	0.13 ± 0.02 ^A^	0.08 ± 0.00 ^B^	0.15 ± 0.02 ^A^	0.13 ± 0.03 ^A^	0.14 ± 0.01 ^A^	0.12 ± 0.04 ^AB^
cTre1	0.15 ± 0.01 ^C^	0.09 ± 0.03 ^B^	0.20 ± 0.02 ^A^	0.16 ± 0.01 ^AC^	0.13 ± 0.00 ^B^	0.17 ± 0.04 ^ABC^
cTre3	0.32 ± 0.13 ^ABC^	0.10 ± 0.04 ^ABC^	0.10 ± 0.03 ^BC^	0.14 ± 0.01 ^A^	0.12 ± 0.01 ^AB^	0.07 ± 0.02 ^C^

Different letters indicate statistically significant differences (*p* < 0.05) between columns.

**Table 4:** Strength (MPa) of edible packaging with orange essential oil and trehalose.

Samples	Silica gel (0%)	MgCl_2_ (32%)	Standard (45%)	NaBr (57%)	NaCl (74%)	KNO_3_ (92%)
OT80	0.08 ± 0.01 ^D^	0.05 ± 0.00 ^C^	0.10 ± 0.01 ^B^	0.03 ± 0.01 ^A^	0.05 ± 0.00 ^C^	0.06 ± 0.00 ^C^
OT20	0.06 ± 0.00 ^A^	0.04 ± 0.01 ^B^	0.07 ± 0.01 ^A^	0.06 ± 0.01 ^A^	0.05 ± 0.00 ^B^	0.05 ± 0.00 ^B^
Tre0.5OT80	0.08 ± 0.00 ^C^	0.06 ± 0.01 ^AB^	0.11 ± 0.02 ^AC^	0.08 ± 0.01 ^ABC^	0.06 ± 0.00 ^AB^	0.05 ± 0.01 ^B^
Tre0.5OT20	0.09 ± 0.01 ^B^	0.06 ± 0.01 ^AC^	0.09 ± 0.02 ^B^	0.07 ± 0.01 ^A^	0.05 ± 0.01 ^C^	0.06 ± 0.00 ^AC^
Tre1OT80	0.11 ± 0.01 ^B^	0.07 ± 0.00 ^AC^	0.10 ± 0.03 ^AB^	0.05 ± 0.02 ^AC^	0.05 ± 0.01 ^C^	0.07 ± 0.00 ^AC^
Tre1OT20	0.07 ± 0.00 ^A^	0.05 ± 0.01 ^AC^	0.09 ± 0.02 ^B^	0.07 ± 0.00 ^A^	0.04 ± 0.01 ^C^	0.05 ± 0.00 ^C^
Tre3OT80	0.24 ± 0.02 ^B^	0.06 ± 0.01 ^AC^	0.20 ± 0.04 ^B^	0.07 ± 0.01 ^A^	0.05 ± 0.01 ^AC^	0.04 ± 0.01 ^C^
Tre3OT20	0.13 ± 0.03 ^C^	0.03 ± 0.01 ^A^	0.08 ± 0.01 ^BC^	0.03 ± 0.02 ^A^	0.05 ± 0.01 ^AB^	0.05 ± 0.00 ^A^

Different letters indicate statistically significant differences (*p* < 0.05) between columns.

**Table 5:** Breaking strain (%) of edible packaging without addition of OEO stored in different humidity conditions.

Sample	Silica gel (0%)	MgCl_2_ (32%)	Standard (45%)	NaBr (57%)	NaCl (74%)	KNO_3_ (92%)
c	66.54 ± 2.85 ^A^	81.25 ± 0.84 ^B^	63.01 ± 1.18 ^A^	10.27 ± 3.92 ^A^	69.95 ± 4.99 ^A^	63.87 ± 1.00 ^A^
cTre0.5	78.71 ± 4.33 ^B^	76.88 ± 3.75 ^AB^	76.13 ± 6.43 ^AB^	69.05 ± 7.29 ^AC^	79.32 ± 2.31 ^B^	65.39 ± 3.97 ^C^
cTre1	72.03 ± 10.16 ^ABCD^	73.08 ± 3.30 ^CD^	71.01 ± 5.42 ^ABCD^	67.40 ± 2.89 ^AC^	77.48 ± 3.35 ^BD^	65.77 ± 2.00 ^A^
cTre3	62.44 ± 1.06 A^C^	62.62 ± 0.37 ^A^	60.87 ± 0.41 ^C^	62.14 ± 0.65 ^AC^	66.18 ± 1.08 ^B^	70.24 ± 3.25 ^B^

Different letters indicate statistically significant differences (*p* < 0.05) between columns.

**Table 6:** Breaking strain (%) of edible packaging with addition of OEO and Tre stored in different humidity conditions.

Samples	Silica gel (0%)	MgCl_2_ (32%)	Standard (45%)	NaBr (57%)	NaCl (74%)	KNO_3_ (92%)
OT80	102.64 ± 2.73 ^C^	99.28 ± 3.26 ^C^	84.22 ± 7.82 ^B^	10.30 ± 8.40 ^A^	74.50 ± 2.42 ^AB^	84.45 ± 3.96 ^B^
OT20	101.65 ± 5.51 ^C^	95.78 ± 4.49 ^AC^	92.42 ± 4.27 ^A^	92.40 ± 3.42 ^A^	83.38 ± 5.60 ^B^	87.41 ± 1.62 ^AB^
Tre0.5OT80	99.64 ± 4.71 ^A^	100.24 ± 6.06 ^A^	69.77 ± 4.30 ^B^	103.39 ± 6.40 ^A^	82.62 ± 1.57 ^C^	85.63 ± 9.46 ^C^
Tre0.5OT20	107.78 ± 5.75 ^B^	108.67 ± 11.39 ^AB^	108.29 ± 2.62 ^B^	95.30 ± 3.81 ^A^	84.21 ± 3.86 ^C^	87.55 ± 2.97 ^AC^
Tre1OT80	95.80 ± 2.95 ^BD^	100.86 ± 5.80 ^B^	72.89 ± 6.53 ^A^	79.18 ± 8.83 ^AC^	74.00 ± 5.78 ^AC^	85.24 ± 1.90 ^CD^
Tre1OT20	93.87 ± 0.85 ^A^	86.43 ± 11.36 ^AC^	111.24 ± 12.71 ^B^	89.26 ± 1.45 ^AC^	79.15 ± 6.63 ^C^	86.82 ± 2.71 ^AC^
Tre3OT80	66.91 ± 0.95 ^BC^	74.75 ± 4.36 ^AC^	64.00 ± 1.65 ^B^	73.83 ± 2.58 ^A^	82.44 ± 5.76 ^AC^	69.48 ± 6.27 ^ABC^
Tre3OT20	73.44 ± 2.26 ^AB^	69.02 ± 2.77 ^B^	74.91 ± 4.23 ^AB^	69.11 ± 9.03 ^AB^	73.95 ± 6.21 ^AB^	75.24 ± 2.32 ^A^

Different letters indicate statistically significant differences (*p* < 0.05) between columns.

## Data Availability

The data used to support the findings of this study can be made available by the corresponding author upon request.
